# Cognitive and affective judgements of syncopated musical themes

**DOI:** 10.2478/v10053-008-0094-0

**Published:** 2011-12-22

**Authors:** Peter E. Keller, Emery Schubert

**Affiliations:** 1 Max Planck Institute for Human Cognitive and Brain Sciences, Germany; 2 School of English, Media and Performing Arts, University of New South Wales, Australia

**Keywords:** syncopation, serial asymmetry, affective response, cognition, rhythm, emotion, musical form

## Abstract

This study investigated cognitive and emotional effects of syncopation, a feature of musical rhythm that produces expectancy violations in the listener by emphasising weak temporal locations and de-emphasising strong locations in metric structure. Stimuli consisting of pairs of unsyncopated and syncopated musical phrases were rated by 35 musicians for perceived complexity, enjoyment, happiness, arousal, and tension. Overall, syncopated patterns were more enjoyed, and rated as happier, than unsyncopated patterns, while differences in perceived tension were unreliable. Complexity and arousal ratings were asymmetric by serial order, increasing when patterns moved from unsyncopated to syncopated, but not significantly changing when order was reversed. These results suggest that syncopation influences emotional valence (positively), and that while syncopated rhythms are objectively more complex than unsyncopated rhythms, this difference is more salient when complexity increases than when it decreases. It is proposed that composers and improvisers may exploit this asymmetry in perceived complexity by favoring formal structures that progress from rhythmically simple to complex, as can be observed in the initial sections of musical forms such as theme and variations.

## INTRODUCTION

Successful composers know how to structure musical materials in order to maintain the listener’s attention and to modulate their cognitive and affective state. One apparent consideration that composers seem to be aware of, we believe, concerns the serial ordering of musical patterns that vary in complexity. There is evidence in psychological, and in particular auditory-perceptual literature that a transition from structurally simple to complex, soft to loud, and consonant to dissonant is more salient than the reverse (loud to soft, etc., e.g., Huron, 1992; Schellenberg, 2001). The present research examined whether there are cognitive and affective implications of creating music that moves from simple to complex or the reverse. To this end, we manipulated a quantifiable aspect of rhythm – degree of syncopation – to create musical materials composed of various serially ordered combinations of simple (and unsyncopated) and complex (and syncopated) melodies. As will be explained in more detail below, *syncopation* is characterised by the emphasis of weak locations in metric structure and de-emphasis of strong metric locations, causing a momentary violation of the listener’s temporal expectancies. For the sake of experimental rigour, we focused on short, specially-composed pieces, and monitored self-reported cognitive and affective responses to changes in the degree of syncopation.

### Complexity and the “theme and variations” form

Instances of improvised or composed music that moves predominantly from simple to complex are abundant.^1^ A ubiquitous musical form that explicitly uses such a compositional approach is the theme and variations. A famous example in the Western classical music literature is the 12 variations on *Ah vous dirai-je, maman* in C, K. 265 (*Twinkle twinkle little star*) by W. A. Mozart. In this piece, the austere theme is exposed and then varied by addition of notes in one variation, changes in texture in another version, changes in the style of the accompaniment, another variation with a countermelody added to the original melody, another variation with the melody played in different registers, and so forth. One variation changes the mode of the tune from the original, which is in a major key, to a minor key. Threaded through the variations are various other subtle and interesting manipulations of musical features. There are clearly noticeable changes in the complexity of the variations, and the rhythm of the melody is frequently manipulated, with syncopated versions appearing in Variations 5 and 11. The contrast between the simplicity of the initial theme and the ornateness of many of the subsequent variations is striking.

In general, the theme stated at the beginning of a piece – whether it employs a theme and variations form or some other structural approach to composition – is in a simple form that tends to get more complex in subsequent manifestations. Understanding whether there may be cognitive preferences that encourage this kind of progression in improvised performance and composition is the broad issue that drives the present research. In addition, we were interested in how emotional responses are influenced by such cognitive preferences related to musical structure, specifically in terms of rhythmic complexity.

### Rhythm and emotion

Music is able to produce emotional expressions that listeners within a given culture can agree upon (e.g., Juslin & Laukka, 2004; Scherer, 2004). This agreement suggests that emotion expressed by music is reasonably reliable and stable (e.g., Bigand, Vieillard, Madurell, Marozeau, & Dacquet, 2005; Gabrielsson & Lindström, 2010; Hevner, 1936; Juslin, 2005; Schubert, 2004). For example, loud, fast music is expressive of high arousal emotions, major mode of happy emotion, and so forth. However, not all relationships between musical features and emotional response are so well established. Rhythm is an important parameter of music, defined in terms of the way that sequences of inter-onset intervals of a group of tones are put together and perceived (London, 2007). Unlike pitch, loudness, and tempo, it is difficult to define *rhythm* operationally as a single parameter that varies in intensity or magnitude. To address this problem, researchers have sometimes quantified rhythms in terms of cognitive, ecological (meaning based), and collative (statistically measurable) variables (rather than physical or psychophysical ones), such as regularity (spanning regular to irregular) and smoothness (smooth to rough; Gabrielsson & Lindström, 2010).

 Gabrielsson (1973) conducted a series of experiments that investigated the perception of rhythm using a range of patterns rated along a series of unipolar descriptive adjective scales. Of interest is his Experiment 6, where 21 monophonic patterns were presented and rated on 59 scales by 22 participants. Among the response items were *simple* and *syncopated* (as translated from Swedish). In a factor analysis of the responses, simple and syncopated items had loadings of opposite signs for each of the four factors reported. The second factor produced the highest absolute value for each of these item loadings (-.81 for simple, and .93 for syncopated). Gabrielsson (1973) reported this factor as “the ‘uniformity-variation’ or ‘simplicity-complexity’ dimension” (p. 255). His analysis suggests that complexity and syncopation are, at least, correlated, and possibly semantically similar. However, the responses were made to a range of rhythms, and an effort was made to vary rhythms along many parameters, rather than specifically controlling syncopation alone. Therefore, it is possible that the relationship between complexity and syncopation was an artefact of this intermixing of rhythmic parameters, rather than due to an independent relationship between complexity and syncopation. 

 The general finding of the Gabrielsson (1973) study was that there are at least three dimensions of rhythm, which can be encapsulated by (a) “simplicity-complexity” or “uniformity-variation”; (b) “energetic-restrained”; and (c) movement character, as in “stuttering-uniform”. The second dimension is most closely associated with emotional responses, whereas the third is associated with technical aspects of the performance. One relationship between a parameter of rhythm and corresponding emotion was pointed out by Gundlach (1935). He reported that “rough”2 rhythms were associated with uneasy emotions, and smooth rhythms with an emotional characterization that was positive (glad, brilliant, flippant, etc.), suggesting a traversal of a valence-related dimension, positive to negative, as rhythms move from “smooth” to “rough”. However, similar terms for this dimension of rhythm have yielded conflicting results: According to the summary by Gabrielsson and Lindström (2010), regular/smooth type rhythms have been associated with adjectives such as happy, serious, dignified, peaceful, majestic, and flippant. On the other hand, irregular/rough rhythms are described (and maybe perceived) as amusing and uneasy. 

 The research does not suggest a clear relationship between valence and the regular/smooth to irregular/rough scale. There is a lack of evidence that this dimension of rhythm is a consistent predictor of an emotional dimension. Nevertheless, the collative “complexity” dimension of rhythm identified by Gabrielsson (1973), which is probably related to the rough-smooth and irregular-regular continua, seems to be the main one that has been explored in past research (for a review, see Juslin & Laukka, 2004). According to our review, previous studies have not supplied a framework capable of predicting how basic physical or psychophysical properties correlate with emotional aspects of rhythm. 

### Syncopation in rhythmic structure

In contrast to the relationship between rhythm and emotion, the relationship between rhythmic structure and cognitive complexity is quite well understood. There is a solid body of research, most of it conducted in the context of Western art music, indicating that syncopated patterns are more structurally complex than unsyncopated patterns in terms of both objective mathematical description as well as subjective perception and production (e.g., Fitch & Rosenfeld, 2007; Longuet-Higgins & Lee, 1982, 1984; Pressing, 1999). According to the majority of approaches, *rhythmic complexity* can be defined according to how well a pattern fits within a metric framework. *Metric frameworks* are cognitive/motor schemas that comprise hierarchically arranged levels of pulsation, with pulses at the “beat level” nested within those at the “bar level” in simple n:1 integer ratios such as 2:1 (duple meter), 3:1 (triple), or 4:1 (quadruple; London, 2004). Metric pulsations are experienced as regular series of internal events, with every n^th^ event perceived to be accented (i.e., stronger than its neighbours).

**Figure 1.**
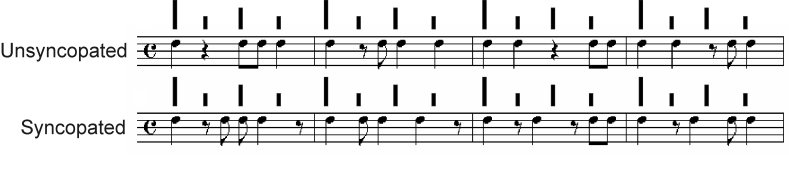
Example of unsyncopated and syncopated rhythm in quadruple meter.

Syncopation occurs when a sound onset coincides with a weak metric location (i.e., between beats) and no sound onset occurs at the next strong metric location (e.g., Jackendoff & Lerdahl, 1983; Johnson-Laird, 1991). Consider the examples shown in Figure 1, wherein each staff contains a rhythmic pattern in quadruple meter (i.e., each bar contains four beats). The pattern notated in the top staff contains rhythmic groups with note onsets coinciding mainly with the beats in each bar. The second staff shows a pattern with a greater incidence of notes occurring off the beats (specifically, half way between them), in addition to situations when no note occurs on a beat, resulting in syncopation even though other musical parameters are held constant across the two patterns (i.e., the number of notes and the pitch stay the same).

 A syncopated rhythm produces a momentary violation of a listener’s (schematic) temporal expectancies (London, 2004), and should therefore evoke emotion because emotion is generated when an expectancy is delayed or inhibited. Specifically, expectancy violation should trigger generalized arousal in the listener, and thus produce a subsequent increase in self-reported arousal (Huron, 2006; Meyer, 1956; Steinbeis, Koelsch, & Sloboda, 2006). The rating of expressed instead of felt emotions should have little impact on the evaluation (Evans & Schubert, 2008), but according to a study by Schubert (2007b) has the advantage of producing more stable responses. 

 We sought to investigate emotional responses associated with listening to syncopated music. In addition to arousal, these responses may include *tension* and *valence*. *Tension* has an ambiguous meaning. It is variously considered to be part of the arousal dimension of the two-dimensional model of emotion proposed by Russell (1979, 1980), a dimension of its own with the label “tension arousal” (Ilie & Thompson, 2006; Schimmack & Rainer, 2002), or a construct that, while closely connected with arousal, has a more musical implication, as in “tension-release” or “tonal tension” (Lerdahl, 1996; Lerdahl & Krumhansl, 2007). Scherer (2005) noted that this latter conceptualization has origins in Wundt’s (1905) three dimensions of valence (positive-negative), arousal (calm-excited), and tension (tense-relaxed). Each of these interpretations of tension may have some distinctiveness, but there are also similarities. If *tension* is semantically identical to *arousal*, then we would expect tension and arousal to be correlated.

By extrapolating from previous studies examining rhythmic complexity and emotional response, we may be tempted to predict that complex/rough rhythms will produce more negative valence emotions (such as sadness, anger, or fear) compared to simple/smooth rhythms (which produce more positive emotion responses, such as happiness). However, it is also possible that such potential effects on valence will be countered by the fact that other features known to affect valence in Western culture – mode (major/minor) and tempo (Gabrielsson & Lindström, 2010; Hevner, 1936) – were held constant in the present investigation. Holding these features constant allowed a strong test of whether variations in syncopation alone influence valence.

### Factors influencing the enjoyment of music

The enjoyment of a piece of music – including the pleasure3 derived from it, a preference for it, or appreciation of its aesthetic value – is modulated by variables such as familiarity (North & Hargreaves, 1997; Schellenberg, Nakata, Hunter, & Tamoto, 2007), perceived emotional content (Gabrielsson, 2010; Ritossa & Rickard, 2004; Salimpoor, Benovoy, Longo, Cooperstock, & Zatorre, 2009; Schubert, 2007a), and complexity (Beauvois, 2007; Berlyne, 1971; Eisenberg & Thompson, 2003; North & Hargreaves, 1998; Orr & Ohlsson, 2005; Schellenberg et al., 2007). Though not always consistent, these studies and reviews reveal that the more emotion a piece of music expresses, the more it is enjoyed (even for negative emotions, see Garrido & Schubert, 2011; Schubert, 1996, 2010); the more familiar it is, the more it is enjoyed; and complexity is most enjoyed when it is reasonably high or at some optimal, moderate level.

In the present study, we attempt to restrict the effects of these variables by examining only two hypothesised levels of rhythmic complexity (syncopated vs. unsyncopated), and by controlling familiarity through the use of novel stimulus melodies in which pitch and rhythmic sequences vary across items. We assume that the use of specially-composed melodies as stimuli will minimise exposure effects upon enjoyment because each item is made up of unique melodic and rhythmic combinations while maintaining the required status of syncopated or unsyncopated.

### Asymmetries in perception

As noted earlier, one of the driving forces behind the current investigation is to examine potential reasons for why musical forms such as theme and variations tend to move from simple to complex, at least initially. We therefore sought to examine whether affective and cognitive subjective ratings are asymmetric – that is, different in magnitude as well as in direction – when moving from syncopated to unsyncopated (i.e., from simple to complex) versus vice versa. Assuming that aesthetic concerns drive preferences for certain musical structures, we might expect that moving from unsyncopated to syncopated patterns will lead to a greater level of enjoyment and heightened emotional charge than when a syncopated pattern precedes an unsyncopated one. Moreover, if these aesthetic and affective responses are governed, at least in part, by cognitive variables, then this asymmetry may be paralleled by an asymmetry in the detection of changes in complexity. If the change from an unsyncopated to a syncopated sequence is more salient than the reverse, then this may provide a cognitive foundation for the aesthetic decision that composers and improvisers make when employing this type of musical progression.

There is ample evidence for perceptual asymmetries in auditory psychophysics. For auditory loudness, looming (a gradual increase in loudness) is known to be more noticeable than the equivalent attenuation of loudness (Neuhoff, 2001; Rosenblum, Wuestefeld, & Saldana, 1993). In music, this effect is consistent with the so-called *ramp archetype* proposed by Huron (1990, 1992). Specifically, Huron has argued that composers of Western art music maintain listeners’ attention by employing intensity increases that are small and incremental, but each followed by stimulus decreases that are large and abrupt, thus forming “ramps” in a work’s intensity profile.

Furthermore, it has been reported that increasing complexity in auditory and musical stimuli is more readily noticed than decreasing complexity. For example, studies by Schellenberg, Trehub, and Trainor (Schellenberg, 2001; Schellenberg & Trainor, 1996; Schellenberg & Trehub, 1996) have found that complex frequency tuning (e.g., a perfect fifth interval departing from 700 cents) and dissonance in pitch intervals are more noticeable if preceded by simpler tuning (perfect fifth equals 700 cents) and less dissonance, than vice versa.

 Studies in the domain of rhythm have yielded evidence of analogous perceptual asymmetries. For example, Bharucha and Pryor (1986) found that listeners were better able to discriminate between auditory patterns that fit an isochronous metric grid and those that did not when the metric pattern was presented as the first item in a pair. Similarly, Handel (1998) found that rhythmic complexity affected discrimination between paired metric patterns only when the simpler pattern was the first of the pair. Accurate discrimination apparently relied upon the use of an unambiguous metric framework that was generated while listening to the initial pattern. Similarly, in the context of theme and variations form or an improvisation, starting with an unsyncopated theme may ensure that such a framework is established and subsequently used as a schema – or perceptual reference frame for pitch/time relations (see Jones, 1990) – to facilitate the perception of the more complex rhythm that follows and to appreciate its syncopatedness. 

### Overview of the current study

The aim of the current study is to examine cognitive and affective responses to changes in rhythmic syncopatedness, and to gauge the cognitive and affective implications of moving from unsyncopated to syncopated for the listener. To this end, we investigated how increases versus decreases in syncopatedness influence cognitive (perceived complexity) and affective (perceived happiness, arousal, tension, and enjoyment) judgements about short tonal melodies. The melodies consisted of two phrases. The rhythm of the first phrase was either syncopated or unsyncopated and the second phrase was either the same or different to the first phrase in terms of degree of syncopatedness. The musically trained participants were required to rate the second phrase of each pattern, relative to its first phrase, with respect to how complex, happy, aroused (excited), and tense it sounded, and how much more or less enjoyable it was. This paradigm was designed to address the following specific research questions:

1. How does a change in syncopatedness affect perceived comple-xity and/or emotional dimensions – namely valence (happiness), activity (arousal) and tension – expressed by musical rhythm?

2. Do listeners enjoy syncopated rhythms more than unsyncopated ones?

3. What subjectively rated emotional and cognitive variables are related to the enjoyment of rhythm?

4. Are there asymmetries in the perception of changes in rhythmic complexity, and are these consistent with asymmetries implied by the convention in musical forms (such as theme and variations) to begin with relatively simple material and then become more complex, rather than the reverse?

## Method

### Participants

Thirty-five upper level undergraduate music students, 19 female and six male, took part in the study in return for course credit. Average age of the participants was 20.8 years (*Mdn* = 20, range 19-33). All participants reported having normal hearing, except one who reported minor hearing loss. This participant’s data were nevertheless retained.

### Stimuli

Melodies consisting of two 4-bar phrases in quadruple meter were used as stimuli. The melodic pitch series repeated across both phrases while the rhythm changed. Four types of rhythmic change were possible: (a) from an unsyncopated rhythm to a syncopated rhythm (US), (b) from a syncopated rhythm to an unsyncopated rhythm (SU), (c) from one unsyncopated rhythm to another unsyncopated rhythm (UU), and (d) from one syncopated rhythm to another syncopated rhythm (SS).

**Figure 2.**
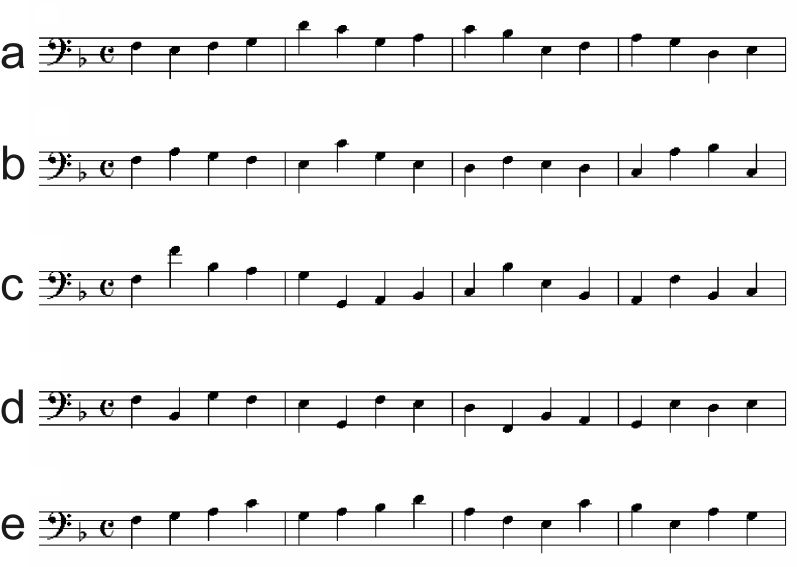
Five melodic pitch series (a-e) from which stimuli were derived.

The stimuli were created by author P.E.K. in several stages. First, five melodious pitch series were composed in the key of F major. These are shown in Figure 2. More than one melody was deemed necessary to reduce the effects of exposure to a particular pitch series (Schellenberg et al., 2007). These melodies were then adjusted rhythmically accor-ding to unsyncopated (U) and syncopated (S) rhythmic templates, as described below.

**Figure 3.**
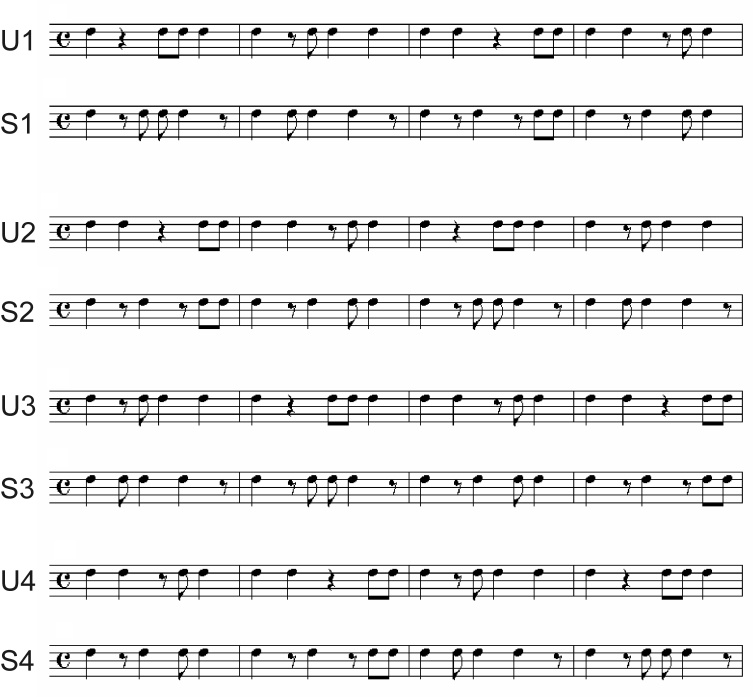
Four unsyncopated rhythmic templates (U1-U4) and four related syncopated templates (S1-S4).

Four U templates were created by concatenating four basic rhythmic motives (each containing four onsets, one on the downbeat, within the space of 1 measure) in various orders determined by a Latin square. S versions of these templates were then created by (a) shifting On-sets 2-4 in two of the measures so that they occurred an eighth note earlier than in the unsyncopated rhythm, and (b) shifting Onset 2 so that it occurred an eighth note late in the remaining two measures. The eight resultant 4-bar rhythmic templates are shown in Figure 3. Notice the prevalence of notes occurring on the strong beat in the U examples, compared to the S examples. The rhythmic templates were then paired in 16 combinations. In four combinations, an unsyncopated rhythm preceded a syncopated rhythm (U1-S4, U4-S1, U2-S3, U3-S2); in another four combinations, these orders were simply reversed (S1-U4, S4-U1, S2-U3, S3-U2); in four combinations, both rhythms were unsyncopated (U1-U4, U4-U1, U2-U3, U3-U2); and in the final four combinations, the rhythms were all syncopated (S1-S4, S4-S1, S2-S3, S3-S2).

Finally, the 16 rhythmic templates and five pitch series were combined exhaustively to yield 80 test stimulus items (with the pitch series repeating across the two phrases of each item). The tonic note (F) with half-note duration was added to the end of each item. In addition to these test items, 20 practice items were created by combining each of the five pitch series with four new rhythmic templates, which were generated by similar rules to those used in generating the test item templates.

**Figure 4.**
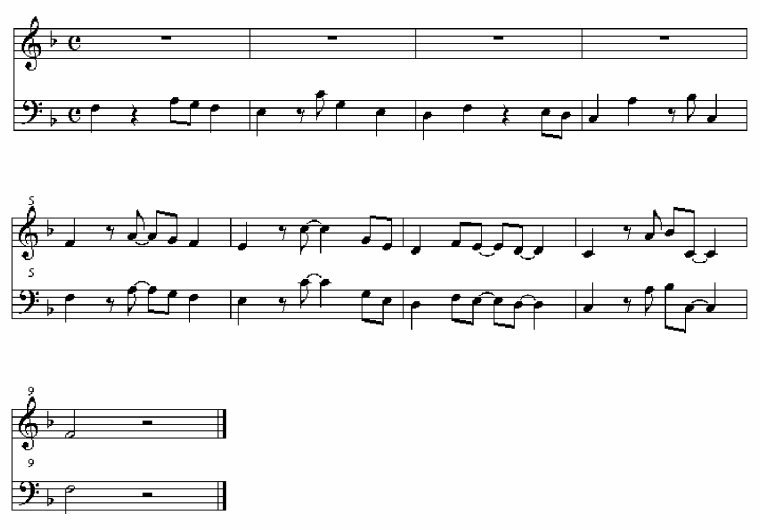
An example test stimulus item showing an unsyncopated theme (bars 1-4) followed by a syncopated variation, accompanied by the addition of a higher instrumental part (bars 5-8).

Stimulus items were stored as MIDI files, which were then played and recorded as .wav files on CD using sampled pizzicato string sounds at a tempo of 120 beats per minute. The first phrase consisted of low strings, which were then joined by high strings at the transition between rhythmic phrases. Thus, the second phrase in each stimulus item was marked by a change in timbre through doubling the melody an octave higher. This was intended to aid the listener in identifying the transition between phrases when making a judgement about the second phrase compared to the first. Note density, duration ratios, pitches, tempo, mode, and nominal intensity (i.e., MIDI velocity) were held constant across stimulus items. A notated example of a test item is shown in Figure 4, where the transition from the unsyncopated theme to a syncopated variation is marked by the addition of a se-cond instrumental part. An item from each of the conditions (US, SU, UU, SS) can be heard in audio examples US14b, SU14b, UU14b, and SS14b (please note that these are not the original sound files used in the experiment).

### Procedure

Participants were tested individually, each sitting at a computer screen and wearing headphones. The software for data presentation and collection was written by author E.S. using Supercard authoring software for Macintosh. After answering background questions and reading ethics approval information, participants began one of five blocks of trials, with each block corresponding to one of the five dimensions being investigated (complexity, enjoyment, happiness, arousal, and tension). In each block participants were given four practice trials followed by 16 test trials (four per US, SU, UU, and SS condition), in which stimuli were presented at a comfortable loudness level. The task was to rate the second phrase of each pattern relative to its first phrase with respect to how complex, happy, aroused, and tense it sounded, and how much more or less enjoyable it was to listen to.

To start each trial, a play button icon was clicked, and after listening, the participant moved a slider to a position on a continuum that was labelled less on the left and *more* on the right, with *no difference* at the middle. As the slider was moved, numerical feedback was provided as a value from -100 to +100 (left to right). Negative values indicated the amount by which the second phrase was lower than the first phrase in the quali-ty referred to by the dimension in question, and positive values indicated the amount by which the second phrase was higher in this quality.

Once a response was made, the participant clicked a right arrow icon to close the current screen and open the screen for the next example. On each screen the following instructions were displayed:

Listen to the melody, comparing the second half with the first half. The start of the second half can be identified by a change in instrumentation (tone colour).

1. Click on the Play button.

2. Rate the second half of the piece with respect to the first half on the scale of:

[Scale name]

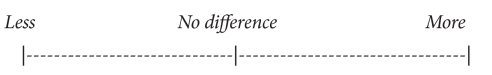

“Scale name” was replaced by one of the five dimension terms, for example *Complexity*, with the exception of *Arousal* for which the text “Arousal (as in ‘Excitement’ or ‘Activity’)” was displayed to reduce the chance of confusion about the term. This dimension name was displayed in large font in each case.

Each of the five blocks (one per dimension) took about 10 min to complete. Participants were encouraged to take a short break after each block. Block and trial order were randomised, and the particular exemplars of test patterns (80 in total) rated with respect to each dimension were counterbalanced across participants. In other words, each participant encountered each of the 80 test items once across the five rating blocks, with the particular set of 16 items encountered in each block being counterbalanced across participants.

### Dependent measures

The main data of interest were ratings from the US and SU test conditions, wherein degree of syncopation changed between melodic phrases. The UU and SS conditions, in which syncopatedness was not varied, were included in the design as baseline conditions to control for response biases (e.g., a tendency to rate the second phrase as higher or lower than the first on the given dimension regardless of syncopatedness). Therefore, our main analyses focused upon indices from which the biases had been partialed out by subtracting each participant’s mean UU and SS rating on each dimension from their corresponding mean US and SU rating. This baseline subtraction also served to remove any effects that the change in timbre (and perceived intensity) between the first and second phrases of the stimulus patterns may have had on ratings.

The above normalization procedure yielded two new indices which are referred to hereafter as US’ (US-UU) and SU’ (SU-SS). The motivation for using these indices is that we were interested in judgements about *changes* in syncopation between phrases (i.e., a relative judgement), not the absolute level of syncopation for the second phrase of each stimulus item. Think of US as an increase in syncopation across the two halves of the stimulus item, SU as a decrease in syncopation, and UU and SS as situations where level of syncopation remains constant throughout the item (low and high, respectively). Following this logic, US-UU is informative about how much the dependent measure in question changes when *syncopation* increases, relative to when is stays at its initial level. SU-SS is informative about how much the relevant dependent measure changes when syncopation *decreases*, relative to when is stays at its initial level.

In addition to participants’ subjective ratings, an objective measure of the degree of syncopatedness in the stimulus items was examined. This objective measure was computed by employing functions from the Matlab MIDI Toolbox (Eerola & Toiviainen, 2004) to analyse the MIDI data that had been used to generate the stimulus items. Specifically, the syncopatedness of each U and S item was quantified by estimating the autocorrelation of note onset times (see Brown, 1993; Toiviainen & Eerola, 2006), which was weighted according to inter-onset interval duration and melodic accent.

Inter-onset interval duration weights were assigned in accordance with Parncutt’s (1994) durational accent model, and melodic accent weights were determined by Thomassen’s (1982) model of melodic accent salience. The total weight assigned to each event was the sum of its durational and melodic accent weights (see Dixon, 2001). Rests were assigned zero weights. Onset times were defined according to the shortest beat-subdivision intervals (i.e., eighth notes, which are half a beat in duration) underlying the rhythmic templates that were described above. The lag-4 autocorrelation of weighted onsets marking these subdivisions – that is, the correlation between the accent strength of events separated by two beats was taken as a measure of syncopatedness. The rationale behind this was as follows: The more similar events separated by two beats are in terms of accent strength, the more the pattern conforms to canonical quadruple metric structure (see Brown, 1993); and, as a corollary, the more different events separated by two beats are in accent strength, the greater the violation of quadruple metric structure. Thus, given our manipulations of metric structure (described above), low lag-4 autocorrelation coefficients are taken to indicate high syncopatedness.

We employed an autocorrelation-based measure with weighted onsets rather than alternative formal methods of estimating rhythmic complexity because we expected that the latter would not be maximally informative in the case of our stimulus patterns. Existing alternative methods (e.g., Fitch & Rosenfeld, 2007; Gómez, Melvin, Rappaport, & Toussaint, 2005; Longuet-Higgins & Lee, 1984; Shmulevich & Povel, 2000) deal only with onset times, relative to an underlying beat or meter, in monotone sequences. They therefore yield identical syncopation scores for all stimulus items within our unsyncopated pool and all items within our syncopated pool (because patterns within pools were constructed from the same basic rhythmic motives). Moreover, alternative methods are designed to handle short cyclic monotone patterns, while we employed longer patterns (with melodic variation) that were suitable for autocorrelation analysis.

The lag-4 autocorrelation coefficient for each U and S pattern used in the study is shown in Table 1. Because we were interested in changes in syncopation from the first to the second phrase of each stimulus item, the lag-4 autocorrelation coefficient for the first phrase was subtracted from the lag-4 autocorrelation coefficient for the second phrase of each US, SU, UU, and SS item. This allowed us to compute US’ (US-UU) and SU’ (SU-SS) indices based on objective measures of syncopatedness, that were analogous to US’ and SU’ based on participants’ judgements. Examining the relationship between objective and subjective measures was intended to permit more fine-grained analysis of how rhythmic structure affects average listener response than what could be achieved by analyses that focus simply on the categorical distinction between syncopated and unsyncopated rhythms. In other words, the correlation analysis aimed at detecting effects of subtle differences in syncopatedness due to melodic and duration accents.

**Table 1.** Lag-4 Autocorrelation Coefficients for Phrases 1 and 2 in Each Stimulus Item (US, UU, SU, SS).

	US		UU		SU		SS	
Stimulus^a^	Phrase 1	Phrase 2	Phrase 1	Phrase 2	Phrase 1	Phrase 2	Phrase 1	Phrase 2
14a	.506	.278	.506	.474	.207	.474	.207	.278
14b	.503	.282	.503	.472	.213	.472	.213	.282
14c	.504	.275	.504	.479	.215	.479	.215	.275
14d	.506	.277	.506	.479	.215	.479	.215	.277
14e	.500	.283	.500	.482	.218	.482	.218	.283
23a	.442	.175	.442	.487	.221	.487	.221	.175
23b	.451	.165	.451	.499	.216	.499	.216	.165
23c	.448	.163	.448	.500	.213	.500	.213	.163
23d	.449	.169	.449	.499	.214	.499	.214	.169
23e	.437	.167	.437	.500	.214	.500	.214	.167
32a	.487	.221	.487	.442	.175	.442	.175	.221
32b	.499	.216	.499	.451	.165	.451	.165	.216
32c	.500	.213	.500	.448	.163	.448	.163	.213
32d	.499	.214	.499	.449	.169	.449	.169	.214
32e	.500	.214	.500	.437	.167	.437	.167	.214
41a	.474	.207	.474	.506	.278	.506	.278	.207
41b	.472	.213	.472	.503	.282	.503	.282	.213
41c	.479	.215	.479	.504	.275	.504	.275	.215
41d	.479	.215	.479	.506	.277	.506	.277	.215
41e	.482	.218	.482	.500	.283	.500	.283	.218
Average	.481	.219	.481	.481	.219	.481	.219	.219

^a^The column lists specific combinations of rhythmic templates (1-4), where the first digit refers to Phrase 1 and the second digit to Phrase 2, and the letter refers to pitch series (a-e).

## Results

### Subjective ratings

**Figure 5.**
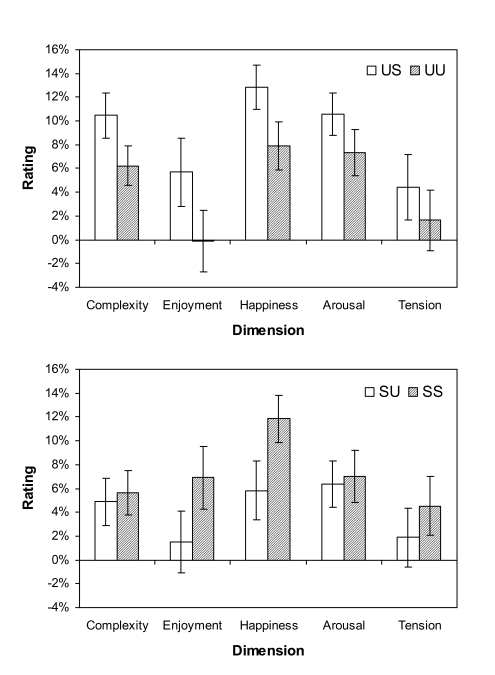
Ratings in US and UU (left panel) and SU and SS (right panel) conditions on the five dimensions. Error bars represent the standard error of the mean.

Ratings for test (US and SU) and baseline (UU and SS) stimuli on each of the five dependent variables, averaged across items and participants, are displayed in Figure 5. The left panel shows ratings in US and UU conditions, and the right panel shows ratings in SU and SS conditions. The values are expressed as percentages of the total range of possible rating values in each direction (i.e., 1 to 100 when Phrase 2 is higher on the rated dimension than Phrase 1; -1 to -100 when Phrase 2 is lower than Phrase 1). The fact that there was an overall positive bias in ratings is quite striking.

**Figure 6.**
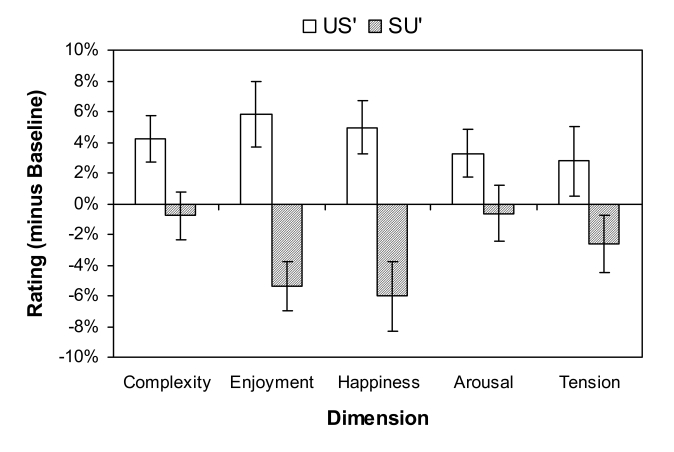
Baseline-corrected ratings (US’ and SU’) for the five dimensions. Error bars represent the standard error of the mean.

As described earlier, response biases and effects of timbre change were partialed out by subtracting each participant’s mean UU and SS rating on each dimension from their corresponding mean US and SU rating to yield US’ and SU’ indices, which are shown in Figure 6. To address the reliability of the effects of increasing (US’) versus decreasing (SU’) syncopatedness on ratings for the five dimensions across participants, US’ and SU’ indices were entered into a 2 x 5 repeated measures analysis of variance (ANOVA) with factors Transition Type (US’ vs. SU’) and Dimension (complexity, enjoyment, happiness, arousal, and tension). The criterion for statistical significance was set at α = .05, and the Greenhouse-Geisser correction was applied when the numerator degrees of freedom exceeded 1. This analysis revealed a statistically significant effect of transition type, *F*(1, 34) = 36.11, *p* < .001, indicating that US’ ratings were reliably higher than SU’ ratings. The main effect of dimension and the interaction between transition type and dimension were not significant, *F*(4, 136) = 0.59, *p* = .66 and *F*(4, 136) = 1.58, *p* = .21, respectively.

We used Fisher’s Least Significant Different (LSD) test to address the hypothesized asymmetries in the perception of increasing versus decreasing syncopatedness. Specifically, participants’ mean ratings for US and UU items and SU and SS items on each of the five dimensions were entered into separate omnibus ANOVAs (US & UU, SU & SS; both of which returned significant results), and then pair-wise comparisons were made between corresponding test and baseline scores (i.e., US vs. UU, SU vs. SS) on each dimension. The outcome of these LSD tests is identical to the results of an analysis in which US’ and SU’ scores for each individual dimension were compared against zero in a series of two-tailed *t* tests (see Table 2). US’ scores on all dimensions apart from tension were significantly greater than zero, indicating reliable increases in ratings (relative to baseline) on these dimensions when syncopation increased. By contrast, SU’ scores were significantly different from zero only for the happiness and enjoyment dimensions, indicating that decreases in syncopation were linked to lower ratings only for these valence-related dimensions. Overall, this qualitative pattern of results suggests that ratings were influenced more strongly by increasing syncopation (which affected four out of five dimensions) than by decreasing syncopation (which affected only two dimensions). Only happiness and enjoyment seem to be immune to this asymmetry.

**Table 2.** The Results of Separate *t* Tests (two-tailed) Against Zero, Including Significance Levels (*p*), for US’ and SU’ Scores on Individual Dimensions.

Condition	Dimension	*t*	*p*
US’	Complex	2,74	.010
	Enjoy	2,67	.012
	Happy	2,80	.008
	Arousal	2,05	.048
	Tension	1,20	.240
SU’	Complex	-0,48	.634
	Enjoy	-3,30	.002
	Happy	-2,67	.012
	Arousal	-0,34	.736
	Tension	-1,41	.168

The next analysis was conducted to examine interrelationships between scores on the five dimensions across stimuli. To this end, intercorrelations were calculated between these dimensions after scores for the 40 (20 US’ plus 20 SU’) items had been averaged across participants. The resultant correlation matrix is shown in Table 3. One of the research questions posed in the Introduction concerned which dimensions are related to enjoyment. As can be seen in Table 3, a significant positive correlation was observed between rated happiness expressed by the musical pattern and enjoyment, indicating that items that attracted high ratings on one dimension also attracted high ratings on the other dimension. None of the other dimensions were correlated reliably with enjoyment. Another research question concerns whether arousal and tension ratings are related. We found no evidence for such a relationship, suggesting that these dimensions were treated independently.

**Table 3.** C orrelation Matrix Showing Interrelationships Among the Lag-4 Autocorrelation (an objective measure of syncopation), Perceived Complexity, Enjoyment, Happiness, Arousal, and Tension Across Stimulus Items.

	Complexity	Enjoyment	Happiness	Arousal	Tension
Autocorrelation	-.239	-.544**	-.627**	-.171	-.316*
Complexity	1	.095	.264	.100	.064
Enjoyment		1	.420**	.048	-.015
Happiness			1	-.033	.132
Arousal				1	-.007
Tension					1

*Note*. *N* = 40. *p < .05. **p < .01.

### Relation between subjective ratings and an objective measure of syncopation

Here we report the results of an analysis of the relationship between participants’ ratings and an objective measure of syncopatedness, which was based on the autocorrelation of weighted note onsets in the stimulus items (see *Dependent Measures* section). First, it can be briefly noted that, as can be seen in Table 1, this objective measure confirmed that conformity to quadruple metric structure (a) decreased from Phrase 1 to Phrase 2 in all US items, (b) increased from Phrase 1 to Phrase 2 in all SU items, and (c) was constant across phrases in all UU items and SS items, on average, while being overall higher for UU than for SS items.

The question of primary interest, however, concerns the relationship between US’ and SU’ indices based on the objective measure and corresponding indices based on participants’ mean ratings. The correlation between objective indices and subjective indices for each dimension was calculated across items to address this issue. As can be seen in Table 3, objective indices were significantly correlated with happiness, enjoyment, and tension, with the negative correlation coefficients indicating that more metric violation (i.e., greater syncopation) attracted higher ratings on these dimensions. Objective indices were not correlated reliably with subjective indices for complexity and arousal.

US’ and SU’ scores were analysed separately in a second set of correlation analyses to address the impact of subtle differences in objective syncopatedness attributable to the effects of melodic and duration accents. Neither of these analyses yielded statistically significant results. This finding indicates that the relationships between participants’ ratings and the objective syncopatedness measure observed in the above analysis of pooled US’ and SU’ scores were driven by the differences in the relative onset times of tones in U and S rhythms, more so than by subtler effects of melodic and duration accents on rhythmic complexity.

## Discussion

The aim of this study was to examine the cognitive and affective responses to musical rhythms that varied in degree of syncopation. We were particularly interested in the cognitive and affective implications of creating music in which rhythmic structure moves from simple to complex or vice versa. Our underlying motivation was related to psychological processes that may drive preferences (by composers, improvisers, and listeners) for simple themes followed by more complex variations, rather than the reverse, in musical forms such as theme and variations. We discuss the results according to the four specific research questions of the study.

1. How does a change in syncopatedness affect perceived com-plexity and/or emotional dimensions namely valence (happiness), activity (arousal), and tension expressed by musical rhythm?

The results of the experiment indicate that perceived complexity increases when a melody moves from unsyncopated to syncopated. However, the reverse is not true: Unsyncopated melodies were rated as statistically equivalent in complexity to syncopated melodies when they followed the syncopated melodies. This asymmetry is discussed in Point 4, below, and provides an explanation of why a reliable overall correlation between syncopation and complexity was not observed.

The results concerning effects of syncopation on affective dimensions were relatively clear. Here it was found that syncopated patterns were rated as happier than unsyncopated patterns, irrespective of the serial ordering of the two types of pattern. Somewhat surprisingly, however, ratings of arousal and tension (which were uncorrelated) indicated weak and generally unreliable effects of syncopation on these dimensions (though the direction of the effects was consistent with the hypothesis that syncopation increases arousal and tension). This may be due to the fact that our patterns contained only moderate levels of syncopation, as is common in much Western classical and mainstream popular music (Huron & Ommen, 2006; Temperley, 2008). The higher the levels of syncopation are and the greater degrees of metric ambiguity (that characterize musical genres such as jazz), the more pronounced impact upon perceived arousal and tension.

Taken together, the current findings suggest that our manipulation of syncopatedness affected valence more than the arousal dimension of emotion. Other works have shown that arousal is strongly modulated by tempo and intensity (loudness) in music (see Dean, Bailes, & Schubert, 2011; Gabrielsson & Juslin, 2003; Schubert, 2004). We held tempo constant, and we controlled for the effects of varying timbre and intensity across the two phrases of our stimuli. If systematic variations in (local or global) tempo and intensity accompany changes in degree of syncopation in natural, live music, then even moderate levels of syncopation may appear to affect arousal and tension “in situ”. However, our results suggest that in the absence of such covariation, syncopation is a device that may primarily be geared towards enhancing positive affect.

2. Do listeners enjoy syncopated patterns more than unsynco-pated ones?

Our results indicate that syncopated rhythms are enjoyed more than unsyncopated patterns. This effect appears to be symmetric and independent of serial order. That is, whether the syncopated rhythm was presented as the first or second pattern within a pair, it was judged to be higher in terms of enjoyment than the unsyncopated pattern (which was rated lower when it was heard as the second pattern of a pair).

Importantly, as was the case with arousal and tension above, we urge caution in drawing a simple conclusion about the effect of syncopation on enjoyment. Our results may be specific to the moderate degrees of rhythmic complexity that characterize the stimuli that we employed. Increasing complexity further (consider, e.g., random inter-onset intervals) may lead to a decline in enjoyment ratings, in accor-dance with theoretical proposals that there is an inverted-U relationship between complexity and factors such as aesthetic preference (Berlyne, 1971; Eysenck, 1968; Fechner, 1897). While some studies question the validity of the inverted-U relationship (Eisenberg & Thompson, 2003; Orr & Ohlsson, 2001, 2005), it is nevertheless possible that our results are limited by the fact that the full range of subjective and stimulus variance was not covered (see Beauvois, 2007). Further research in which rhythmic complexity is extended to levels that may reduce preference responses could lead to a more complete understanding of the relationship between cognitive processing and aesthetic response.

3. What subjectively rated emotional and cognitive variables are related to the enjoyment of rhythm?

While the enjoyment of syncopation is a major finding of our study, we also sought to investigate whether enjoyment is related to other subjective variables. This question was motivated by the fact that previous studies have highlighted the importance of emotion as a contributor to musical preference (see Schubert, 2007a). Our design allowed this issue to be investigated in the context of musical rhythm.

We found that, overall, perceived complexity, arousal, and tension ratings did not correlate significantly with enjoyment across items within our pool of stimulus items, but happiness ratings did. Syncopated rhythms were enjoyed more and considered happier than unsyncopated rhythms, regardless of whether the syncopated pattern appeared first or second in the stimulus pair. Although the finding that happy sounding rhythms are enjoyable is unsurprising, the fact that happiness was the only factor that was related to enjoyment is noteworthy insofar as it supports our earlier proposal that syncopation functions primarily to enhance positive affect.

4. Are there asymmetries in the perception of changes in rhythmic complexity, and are these consistent with asymmetries implied by the convention in musical forms (such as theme and variations) to begin with relatively simple material and then become more complex, rather than the reverse?

We predicted an asymmetric effect of changes in syncopatedness on perceived complexity on the basis of auditory psychophysical work and the apparent predominance of increasing complexity in musical forms such as theme and variations. It was an open question whether asymmetries in enjoyment, happiness, arousal, and tension would be observed.

Our results indicate that valence-based assessments (enjoyment and happiness) are symmetrical in serial order: Movement from a simple to a complex rhythm produced high (positive) ratings to the same degree that movement from a complex to a simple rhythm produced relatively low (negative) ratings. The perception of tension was similarly symmetric, though increases and decreases on this dimension were not statistically significant.

 The cognitive variable of perceived complexity, however, showed strong serial order asymmetry, as expected. Melodies that moved from simple (unsyncopated) to complex (syncopated) were reported to increase in complexity, while no statistical change in complexity was reported in the case of complex to simple progressions. Such an asymmetry was also observed in arousal judgements, though the effect was weak. The superficial similarity of arousal and complexity ratings may suggest that perceived complexity (but not necessarily objective complexity) is linked more strongly to emotional arousal than to valence.^4^ This is in agreement with the results of Timmers and Ashley (2007), who reported that ratings of low arousal emotions of sadness and love were negatively correlated with complexity in a flute performance with different types of ornamentation. High arousal emotions of happiness and anger, on the other hand, were positively correlated with com-plexity in their study. 

Overall, our results point to a dissociation between complexity ratings (which were asymmetric with regard to serial order) and happiness/enjoyment ratings (which were symmetric). This dissociation can be taken to suggest that preferences for serial progressions that move from simple to complex materials in music at least in the case of rhythm may stem more from cognitive considerations related to perceived complexity than from affective considerations, such as perceived valence.

We speculate that composers and improvisers may intuitively favor musical forms characterized by progression from structurally simple to complex rhythmic materials for two reasons. First, unsyncopated rhythms allow cognitive/motor schemas such as metric frameworks to be readily invoked and used to facilitate the perceptual and cognitive processing of the relatively complex syncopated rhythms that follow. Second, the serial ordering of complexity relations may influence the salience of structural changes, and thus shape their aesthetic implications. On this note, our finding that changes from unsyncopated to syncopated patterns influenced perceived complexity, while the reverse was not the case, suggests that increasing syncopa-tedness is more salient than decreasing syncopatedness. It is possible that the degree to which a rhythmic pattern engages motor-related areas of the listener’s brain increases with increasing complexity (Chen, Penhune, & Zatorre, 2008; Engel & Keller, 2011). Thus, syncopation may enhance the processes of internal sensorimotor simulation and online prediction that accompany music listening, and thereby promote aesthetic enjoyment (Kornysheva, von Cramon, Jacobsen, & Schubotz, 2010).

In accordance with this conceptualization, changes in rhythmic structure that progress from unsyncopated to syncopated are especially salient and aesthetically valuable by virtue of the fact that they engage the listener’s motor system relatively strongly. In other words, increases in rhythmic complexity *move the listener* to a greater degree than decreases in complexity. Our results suggest that such decreases have negligible effects on perceived complexity, and they in fact reduce enjoyment. This state of affairs may encourage composers and improvisers to adopt formal conventions in which complexity is increased incrementally over the course of a work’s large scale structure, while decreases in complexity (which are necessary to create contrast and to maintain optimal, moderate global levels of complexity in a work) are less frequent and more abrupt. Thus, our results suggest that the concept of the ramp archetype (Huron, 1990, 1992) may apply to rhythmic complexity, and specifically the treatment of syncopation.

## Conclusions

The findings of the current study suggest that the serial ordering of rhythm patterns that vary in complexity (unsyncopated to syncopated vs. syncopated to unsyncopated) influences the perceived complexity and emotional content of music. Whereas the enjoyment and perceived happiness of musical rhythms are modulated symmetrically with increases and decreases in syncopation between short musical phrases, perceived complexity is heightened with increasing syncopation but remains constant in the face of decreasing syncopation. This asymmetry in perceived complexity (which also characterizes perceived arousal to some degree) has implications for the cognitive processing and aesthetic appreciation of musical rhythmic structure. Successful composers and improvisers may be sensitive to these implications, and consequently favor musical forms in which progression from simple to complex material is more prevalent than the reverse.

A final remark on the generalizabilty of our findings and, more broadly, the universality of musical cognitive and emotional processing is in order. The fact that our study employed a set of rhythmic stimulus materials that were restricted to a single meter (quadruple), tempo (120 beats per minute), and mode (F major), raises the question whether similar results would be observed with different materials. It would, in future work, be particularly interesting to compare the perception of changes in rhythmic complexity in Western musical traditions and in cultures where rhythm is organized by principles other than metric hierarchies built on simple integer ratios (e.g., Indian alap, African polyrhythm, and Balkan folk music; see Arom, Thom, Tuckett, & Boyd, 1991; Chernoff, 1979; Clayton, Sager, & Will, 2005; Hannon & Trehub, 2005; London, 2004). Such cross-cultural comparisons are potentially informative about musical universals for example, processes related to basic perceptual and cognitive constraints (Stevens & Byron, 2009) and to the recognition of basic emotions (Fritz et al., 2009) as well as in highlighting the rich diversity in human music-making (Becker, 2009; Clayton, 2009; Nettl, 2005).

## Footnotes

1 Note that there are typically local (e.g., within-phrase) fluctuations in complexity throughout a work even when its complexity increases globally (e.g., between phrases and sections).

4 It should be noted that this holds for listeners’ arousal ratings averaged over stimulus items within US’ and SU’ pools, but not for the correlation analysis of averaged listener ratings across all stimulus items (perhaps due to low variance in arousal ratings between items).
